# Analysis and Assessment of Exposure to Selected Phthalates Found in Children’s Toys in Christchurch, New Zealand

**DOI:** 10.3390/ijerph15020200

**Published:** 2018-01-25

**Authors:** Matthew James Ashworth, Andrew Chappell, Ellen Ashmore, Jefferson Fowles

**Affiliations:** 1 Institute of Environmental Science and Research (ESR) Ltd., Christchurch 8041, New Zealand; Andrew.Chappell@esr.cri.nz (A.C.); Ellen.ashmore@esr.cri.nz (E.A.); 2 Tox-Logic Consulting, LLC, Petaluma, CA 94954, USA; tox-logic@hotmail.com

**Keywords:** phthalate, exposure, children, toys, mixture, hazard index

## Abstract

Internationally several phthalates are subject to regulatory control regarding maximum allowable concentrations in children’s toys. Such regulation is not in place in New Zealand. Phthalates have been associated with developmental toxicity and endocrine disruption. We determined the concentration of seven phthalates in children’s toys purchased in Christchurch, New Zealand. These results provided data for an exposure assessment deriving Hazard Indices (HI) for oral and dermal exposure routes in children, based on the concentration of mixtures of phthalates shown by the EU to produce either reproductive/developmental or hepatotoxic effects. Of the 49 toys analyzed, 65% contained at least one phthalate at a concentration of >0.1% by mass; and 35% contained multiple-phthalates at individual concentrations of >0.1%. A HI of 3.4 was derived for the combined exposures to the four phthalates associated with reproductive and developmental effects. A HI of 0.3 was derived for the group of phthalates associated with hepatotoxic effects. Five phthalates were detected at levels exceeding the EU regulatory limit of 0.1% by mass. Risk assessment calculations indicate that, using realistic exposure scenarios, the worst-case combined exposure to phthalates associated with developmental toxicity exceeded a HI of 1 so may cause adverse developmental effects.

## 1. Introduction

Phthalates are chemical additives to plastics and polymers to increase physical flexibility. They are commonly used in polyvinyl chloride (PVC) plastics, which occur in a range of materials and objects found in the home, including children’s toys [1,2]. Phthalates are associated with the materials they are added to, and not bonded chemically to them, allowing them to migrate from the source materials into the environment over time [3,4,5,6,7].

Toxicological properties of phthalates include endocrine effects on androgen sensitive tissues, neurological effects, and hepatotoxic effects [8,9,10,11,12,13]. Developmental toxicity has been well characterized in rodents, in the form of lowered testes weight, shortened anogenital distance, and lower testosterone production [10,13,14,15,16]. A longitudinal study by Huang and associates [10] found that urinary metabolites of DEHP are associated with decrements in neurological development in children exposed to phthalates post-natally. Diisononyl phthalate (DINP), di-n-octyl phthalate (DNOP) and diisodecyl phthalate (DIDP), on the other hand, are all noted to produce hepatotoxicity in the rat, through peroxisome proliferator receptor mechanisms that have questionable relevance to humans [8,9,11,17].

The co-exposure to multiple phthalates with common modes of action has resulted in researchers using a hazard index (HI) approach to capture the combined toxicological risk of similar phthalates. Chang and associates examined the cumulative dietary intake of groups of phthalates in Taiwanese children of varying age, and found that the HI for 5 anti-androgenic phthalates was >1.0, at the 95th percentile of estimated intake [18]. On a population level, individual phthalate exposures and risks in the U.S. have also been assessed using a HI approach with urinary phthalate biomonitoring data obtained from the National Health and Nutrition Examination Survey (NHANES) [19]. In their study, Christensen et al. [19] found median DEHP levels to exceed a hazard index of 1.0 for all age groups, but most significantly in ages 6–11 years.

There are multiple exposure pathways for phthalates to infants and toddlers through their diet and other environmental routes [20]. Outside the home, this group receives reported exposures to phthalates while in cars, preschools and daycare centers [21,22,23,24,25,26]. Direct dermal contact with phthalate-containing materials and inhalation or oral intake of household dust are also significant routes of exposure [27,28,29,30,31,32].

International regulations exist for the concentration of phthalates in children’s toys. Regulations from Argentina, Brazil, Canada, the European Union, and the USA provide for a maximum permitted level of 0.1% by weight. Individual phthalates included in these regulations are DEHP, BBP, DBP, DNOP, DINP, and DIDP. The permitted levels of these phthalates in children’s toys and other childcare products is presented in Table 1.

The 0.1% limit in toys, either as total of the listed individual phthalates, is intended to prohibit the functional application in PVC plastic. A minimal level of 10% phthalates, by weight, is required for the softening effect on PVC. The regulatory limit of 0.1%, or even 1% (as in Australia), effectively precludes the use of phthalates for their intended physical function. Consequently, these limits are not based on health hazard or risk [17].

The purpose of this study was to carry out small scale, local survey of concentrations of seven phthalates in toys for infants and toddlers. Toys were purchased from retail outlets in Christchurch, New Zealand. The phthalates selected for analysis were the same as those identified and regulated in several national and regional jurisdictions around the world.

## 2. Materials and Methods

To ensure that best practice was adhered to, each sample was analyzed in an ISO17025 accredited laboratory according to the ISO8124—Part 6:2014 standard for the determination of content of BBP, DBP, DINP, DNOP, and DIDP in children’s toys and plastic children’s articles.

The European Union DG Health and Consumer Product, Public Health Division [33] have proposed tolerable daily intakes (TDIs) for phthalates. Table 2 lists the proposed TDIs, and includes the DIBP acceptable daily intake (ADI) from the US Consumer Product Safety Commission [34].

Sample collection: 49 samples of colored plastic children’s toys were purchased from retail outlets in Christchurch, New Zealand. Toys were chosen on the basis of their physical properties, predominantly selecting items constructed wholly or partially using soft plastic materials. Samples were stored at room temperature in their original packaging until sample preparation.

Analysis: Glassware cleaning procedure: All glassware was cleaned by soaking overnight in a solution containing phosphate-free cleaning agent (Decon 90). The glassware was then rinsed with tap water to remove the cleaning agent, rinsed with de-ionized water, and left to air dry. Before analysis, all glassware was rinsed with acetone and dried thoroughly.

### 2.1. Analytical Methods

Each toy sample was analyzed for seven phthalates according to International Standard ISO 8124-6:2014(E). These phthalates were: DIBP, DBP, BBP, DEHP, DNOP, DINP and DIDP. Briefly, a representative portion of the toy was cut into pieces no larger than 5 mm in any dimension. Approximately 1 g of test portion was accurately weighed and added to a cellulose extraction thimble. Cotton wool was added to each extraction thimble to stop the sample from floating. Each test portion was extracted with 120 mL of dichloromethane for 6 h using soxhlet apparatus. After extraction, the volume of dichloromethane was reduced to approximately 10 mL using a rotary evaporator. The extract was transferred to a 25 mL volumetric flask along with 1 mL of the internal standard (IS) stock solution (250 mg/L Diamyl phthalate (DAP), AccuStandard Inc., New Haven, CT, USA), and made up to the mark with dichloromethane. A portion of dichloromethane solution (containing phthalates) was transferred to a vial for analysis.

### 2.2. Instrumental Analysis

The seven phthalates and internal standard were measured using a gas chromatograph (Shimadzu GC2010, Shimadzu Scientific Instruments (Oceania) Pty Ltd.; Auckland, New Zealand) coupled with a mass spectrometer (Shimadzu GCMS QP-2010 Plus, Shimadzu Scientific Instruments (Oceania) Pty Ltd.; Auckland, New Zealand) run in selective ion monitoring mode. Separation was achieved using a fused-silica capillary column (J&W DB-5MS (Agilent, Wilmington, DE, USA); 30 m × 0.25 mm i.d. × 0.25 μm film thickness). Samples (1 μL) were injected in split mode (split ratio 20.0). The temperature of the injection port was 300 °C, and the detector was 230 °C. The oven temperature was programmed from 80 °C (held for 0.5 min), raised to 300 °C over 25 min, and held at the final temperature for 10 min. Ion fragments 149 m/z (quantifier) and 223 m/z (qualifier) were monitored for DIBP and DBP, 149 m/z and 206 m/z were monitored for BBP, 149 m/z and 279 m/z were monitored for DEHP, 279 m/z and 261 m/z were monitored for DNOP, 293 m/z and 127 m/z were monitored for DINP, 307 m/z and 141 m/z were monitored for DIDP. DAP was used as the internal standard, with the ion fragments 149 m/z and 237 m/z monitored.

DIBP, DBP, BBP, DEHP and DNOP were quantitated against the internal standard DAP as specified in ISO 8124-6 [35], with the standard curve ranging from 0.4 mg/kg to 10 mg/kg. DINP and DIDP are mixtures of esters of o-phthalic acid, and were quantitated as a whole group against the internal standard DAP with the standard curve ranging from 2 mg/kg to 50 mg/kg.

Quality control: The limit of quantitation (LOQ) was 0.001% for DBP, BBP, DEHP and DNOP. The LOQ was 0.005% for DINP and DIDP.

A method blank and a spiked blank were extracted and run with each batch to check for contamination and instrumental performance. The blanks were extracted and analyzed in exactly the same manner as for toy samples. The spiked blank was prepared by adding 1 mL of 100 mg/L stock solution of phthalates in the method blank. No matrix spikes were required for compliance with the ISO8124-6:2014.

Exposure assessment: We adopted the National Academy of Sciences (NAS) approach to assessing cumulative risk for exposure to phthalates [36]. The NAS approach assumes effect additivity when determining benchmark dose estimates for phthalates. Additional assumptions regarding the proportions of individual phthalates. Table 2 illustrates the numerical values used to determine a combined TDI for exposure to phthalate mixtures. Behaviours relevant to the exposure assessment, such as infant and toddler mouthing frequency, were adopted from the CHAP report appendices [17] and from the National Industrial Chemical Notification and Assessment Scheme (NICNAS) reports on DBP, DINP, and DEHP [37,38,39].

Exposure scenarios: The maximal, mean, or median concentrations found in the survey of children’s toys in this study were used to develop exposure scenarios for each phthalate. This approach is supported by the findings in the European Union Rapid Alert System for dangerous non-food products (RAPEX) database [40].

Oral exposures: Estimates of oral exposure to phthalates from mouthing behavior of toys were adapted from the USEPA [41] mean and 5th percentile of child bodyweight, duration of mouthing behavior, and estimated chemical migration rate from the toy [37,42,43]. The exposure estimates were based on a six month old child, as this age group has been shown to exhibit maximal mouthing behavior combined with a low body weight.

Child body weights of 7.4 kg (mean), or 5.7 kg (5th percentile), were considered. The lower (5th percentile) weight provides a more conservative exposure assessment. The body weight data used represented mean of both sexes [41].

An estimate of 10 cm^2^ was used to approximate the typical surface area of an article available for mouthing in a given setting [44]. A worst-case estimate of 2.2 h per day and a more typical duration of mouthing behavior was 0.8 h per day [37,43].

The rate of migration of phthalates from the polymer matrix (*M*, in Supplementary Materials Equation (S1)) is adopted from the data of Chen [45], which reported results of 10 volunteer adults chewing PVC material of approximately 10.3 cm^2^ surface area, with a DINP concentration of 43%. The chewing was done over four 15-min sessions, with saliva collected after each session and analyzed for phthalate concentration. The resulting DINP concentrations ranged from 6.14 to 57.93 µg/cm^2^/h. The mean rate of migration was determined to be 26.03 µg/cm^2^/h. The mean migration rate from the Chen study was used in our exposure assessment. The oral bioavailability of phthalates in our exposure assessment was assumed to be 100%.

For each phthalate in this study, an internal dose was calculated as presented in Supplementary Materials Equation (S1). The compositional % of individual phthalates from each toy or childcare product was accounted for using a modifying factor (*R_phth_*) as shown in Supplementary Materials Table S1. The maximum, mean, and median concentration values were used to calculate the dose from each phthalate. Those phthalates with anti-androgenic modes of action (DEHP, DBP, DIBP, and BBP) formed one grouped toxicological category, whereas DINP, DNOP, and DIDP formed a group with hepatotoxicity as the primary effect. The combined contributions of each phthalate in these groups were calculated assuming effect additivity [46]. Supplementary Materials Equation (S2) shows the calculations of individual hazard quotients (HQ) based on specific phthalate TDIs or ADIs. Hazard quotients were derived from dividing the calculated dose concentration by the TDI or ADI (reference dose) for the specific compound. A calculated HQ value of ≥1 indicates that the exposure may cause adverse effect from a lifetime exposure, but normally this would be from an HQ several times over unity. A value of <1 indicates no adverse effects would be expected over a lifetime exposure. The overall HI was derived from the sum of individual phthalate HQs for each toxicological effect.

Dermal exposure: The child’s exposure to phthalates from direct skin contact was assumed to occur primarily from hand and lip contact from holding and mouthing toys, proportional to the duration of the behavior and the exposed surface area. We employed the 5th percentile of body weight for 6 months old children to conservatively estimate the dose from direct skin contact with toys containing phthalates.

The rate of dermal absorption was estimated from limited quantitative data from DEHP studies. In their study, Deisinger and associates reported on the dermal absorption of DEHP from PVC polymers in rats [47]. PVC sheets (15 cm^2^; 40% DEHP by weight) were placed in contact with shaved skin of 8 male rats. The mean absorption of DEHP was 0.24 µg/cm^2^/h in rats.

In vitro dermal absorption studies have shown that the rate of DEHP absorption is approximately 4 times higher than in humans [48,49]. While comparative in vivo data are not available, the conclusion that rats serve as a conservative model for human dermal absorption is supported by the existing data.

A dermal absorption rate of 0.24 µg/cm^2^/h was used in all dermal exposure estimates in this report. Child body weight values used were 7.4 kg (mean) or 5.7 kg (5th percentile) [17,41]. The amount of time spent by a child touching toys was assumed to be 2.2 h per day and a typical amount of time was 0.8 h per day [50,51]. The contact surface area was 100 cm^2^ for both hands and lips [37]. The internal dose for a 6-month old child from the dermal route was calculated using Supplementary Materials in Equation (S3) and the individual phthalate HQs are shown in Supplementary Materials Table S2.

## 3. Results

Phthalate concentration in samples. Forty-nine samples were analyzed for each of the seven phthalates identified. Of the 49 samples analyzed 32 contained one or more phthalates at a concentration greater than 0.1% by mass.

The frequency of detection of phthalates in the selected toys varied, the highest occurrence being for DIDP, present in 41% of the samples analyzed; only one phthalate, BBP, was not detected above 0.1% by mass in any of the samples. The highest concentration reported for an individual phthalate was for DEHP, at 54% by mass of the sample analyzed. The distribution of phthalate concentrations was not uniform up to the maximum reported, rather the predominant concentrations seen lay in the 0.1–1.0% by mass range. The exception to this was for DEHP where the greatest number of positives reported lay in the range 10.1–20% by mass (6 samples), followed by 30.1–40% (5 samples). The maximum, mean and median reported concentrations are shown in Table 3.

### 3.1. Exposure Assessment

Exposures were calculated for each of the phthalates analyzed, both for oral (Table 4) and dermal (Table 5) exposure routes. From these exposure data HQs were determined for the maximum, mean and median concentrations reported. The greatest reported dose received via both oral and dermal exposure was from DEHP. However, due to the difference in TDIs for the compounds the highest HQ was recorded for DBP under maximum exposure conditions. When considering the mean exposure concentration DBP and DEHP returned the highest HQ values.

### 3.2. Risk Characterization

The HI’s for the phthalates with internationally recognized reproductive or developmental toxicity via anti-androgenic mode of action are shown in Table 6. The HI values are the combined sum of individual HQs for each of the phthalates. Oral and dermal routes of exposure are separately considered and shown in Table 6, based on maximum, mean, and median phthalate concentrations. The absorption of oral phthalates was assumed to be 100%, hence the external dose is equivalent to internal dose for the oral route.

The phthalates identified as hepatotoxic were assessed together and individual HQ values under exposures conditions as described in the exposure scenarios.

The HI values obtained by summing the HQ values are given in Table 7. The HI values represent the highest individual phthalate concentration found in this report and include both ingestion and dermal exposure routes to DINP, DIDP and DNOP.

## 4. Discussion

This report found highly variable phthalate concentrations in children’s toys, with the highest measured % by mass (54.1% for DEHP) being over 500 times the allowable limit in many countries.

Elevated exposures to DEHP and DBP have been shown in recent studies to be negatively associated with impaired neurological development, particularly language learning and expression, in pre-school and school aged children [52]. Table 6 and Table 7 show the potential health risk from exposure to the maximum, mean, and median reported phthalate concentrations from oral and dermal contact with the toys analyzed in this study. These exposure assessments represent the concentrations reported from all samples (toys) analyzed. Hence it should be noted that achieving the level of exposure indicated in this assessment would be likely to require access to a number of different toys.

Dietary exposure to phthalates was not included in the current assessment. However, it should be recognized that the exposure of infants and toddlers to phthalates due to contact with toys is unlikely to be the principal exposure route for phthalates. The primary route of exposure for most phthalates, including those discussed in this report, (but excluding DNOP), is through the diet. Childcare products represent the primary exposure route for DNOP [17]. Thus, the actual total daily exposure to the phthalates described in this report, would be expected to substantially exceed the doses estimated from toys alone. However, the toys appear to be a significant and potentially manageable contributor to daily phthalate exposure in the household/domestic environment.

The HI derived for the anti-androgenic phthalates group (Table 6) represents an exposure which, under worst case scenarios, may be sufficient to produce adverse effects from chronic exposure. Our study has shown that the combined exposure to anti-androgenic, reproductive/developmentally toxic phthalates, can exceed an HI value of 1.0 and thus hold the potential to produce adverse effects upon chronic exposure based on the worst case scenario for infants of 6 months of age. The exposures described in this report are likely to be underestimates of total daily intake, since we did not include addition from other known sources including diet, other indoor materials, or personal care products [17]. However, balanced against these factors is the conservatism from assuming that infants will be exposed to the maximum observed concentration for all phthalates. In addition, it should also be noted that exposure via the scenarios and routes proposed for these assessments will most likely not be lifelong. However, they are occurring during a time of critical development.

When considered in isolation from other phthalate exposure sources, the HI derived for the hepatotoxic phthalate group represents an exposure from toys which is unlikely to produce any adverse hepatic effects from a chronic exposure as it is significantly less than the current TDI. The combined exposure to hepatotoxic phthalates from children’s toys did not result in an HI value (0.3) that would indicate a potential of producing adverse effects. However, this conclusion also is limited by the fact that the contribution of daily phthalate intake from other sources needs to be considered.

This set of 49 samples is a limited, non-random study of the plastic toy products available in New Zealand. A more representative study would require a larger sample size and randomization of sample selection. However, the finding of high percentages by mass of phthalate in some of these samples shows that exposures for children of potential health significance could occur.

## 5. Conclusions

The concentration of seven phthalates in children’s toys purchased in Christchurch, New Zealand was determined. These results provided data for an exposure assessment deriving Hazard Indices (HI) for oral and dermal exposure routes in children, based on the concentration of mixtures of phthalates shown by the EU to produce either reproductive/developmental or hepatotoxic effects. Of the 49 toys analyzed, 65% contained at least one phthalate at a concentration of >0.1% by mass; and 35% contained multiple-phthalates at individual concentrations of >0.1%. A HI of 3.4 was derived for the combined exposures to the four phthalates associated with reproductive and developmental effects. A HI of 0.3 was derived for the group of phthalates associated with hepatotoxic effects. Five phthalates were detected at levels exceeding the EU regulatory limit of 0.1% by mass. Risk assessment calculations indicate that, using realistic exposure scenarios, the worst-case combined exposure to phthalates associated with developmental toxicity exceeded a HI of 1 so may cause adverse developmental effects. The HI derived for hepatotoxic phthalates was below the 1, so, in isolation would not be considered to represent a risk of harm from a lifetime exposure. It is noted in this paper that these exposures represent only one source amongst a host of other, largely better recognized and significant exposure sources.

## Figures and Tables

**Table 1:** Examples of national regulatory limits for phthalate composition of children’s toys.

Country or Region	Maximum Permitted Levels (w/w%)
Argentina	Children’s toys and daycare items for children <3 years old: DEHP (Di (2-ethylhexyl) phthalate) + BBP (Benzbutyl phthalate) + DBP (Dibutyl phthalate) <0.1% For articles that can be placed into a child’s mouth: DEHP + BBP + DBP + DNOP (Di-n-octyl phthalate) + DINP (Diisononyl phthalate) <0.1%
Australia	Children’s toys and daycare items for children <3 years old: DEHP <1%
Brazil	Ethylvinyl toys and childcare articles: DEHP, BBP, DBP <0.1% Ethylvinyl toys and articles that can be placed in mouth of children <3 years old: DEHP, BBP, DBP, DNOP, DINP <0.1%
Canada	Ethylvinyl toys and childcare articles: DEHP, BBP, DBP <0.1% Soft ethylvinyl toys and articles that can be placed in mouth of children <4 years old: DNOP, DIDP (Diisodecyl phthalate), DINP <0.1%
European Union	All children’s toys or childcare articles for children <3 years old: DEHP + BBP + DBP <0.1% All toys and childcare items that can be placed in a child’s mouth: DIDP + DINP + DNOP <0.1%
Japan	All synthetic polymer toys: DEHP is prohibited For all mouth contact toys for children under 6 years old: DEHP and DINP are prohibited
United States of America	For toys of children under 12 years old that cannot be mouthed: DEPH, BBP, DBP <0.1% For mouthed toys of children under 12 years old: DEHP, BBP, DBP, DIDP, DINP, DNOP <0.1%

**Table 2:** European tolerable daily intake (TDI) values for individual phthalates.

Phthalate	Toxicological Target	TDI (µg/kg bw/day)
DEHP ^1^	Reproduction	50
DBP ^2^	Development and reproduction	10
BBP ^3^		500
DIBP ^4^		100
DINP ^5^	Liver	150
DIDP ^6^		150
DNOP ^7^	Liver and thyroid	None available

^1^: Di (2-ethylhexyl) phthalate; ^2^: Dibutyl phthalate; ^3^: Benzbutyl phthalate; ^4^: Diisobutyl phthalate; ^5^: Diisononyl phthalate; ^6^: Diisodecyl phthalate; ^7^: Di-n-octyl phthalate.

**Table 3:** Phthalate concentration ranges in children’s toys and maximum values.

% Phthalate by Mass	DIBP	DBP	BBP	DEHP	DNOP	DINP	DIDP
0–0.1	42	45	49	32	47	35	29
0.1–1.0	5	2	-	1	2	7	19
1.1–10	1	-	-	1	-	3	1
10.1–20	-	2	-	6	-	3	-
20.1–30	1	-	-	2	-	-	-
30.1–40	-	-	-	5	-	1	-
40.1–50	-	-	-	1	-	-	-
50.1–60	-	-	-	1	-	-	-
60.1–100	-	-	-	-	-	-	-
total sample number	49	49	49	49	49	49	49
% of samples > 0.1% Phthalate	14.3	8.2	0.0	34.7	4.1	28.6	40.8
Max. concentration (% by mass)	27.6	15.4	0.031	54.1	0.041	32.3	1.02
Mean concentration (% by mass)	1.71	1.53	0.01	8.62	0.04	6.20	0.25
Median concentration (% by mass)	0.040	0.030	0.014	0.010	0.011	0.78	0.17

**Table 4:** Calculated dose (µg/kg bw/day) and HQ for oral exposure to phthalates at maximum, mean and median exposure concentrations.

Calculated Dose and HQ for Oral Exposure	DIBP	DBP	BBP	DEHP	DNOP	DINP	DIDP
D*_int,oral Bwmean, max_* (Internal dose, oral exposure, mean bodyweight, maximum reported Phthalate concentration)	18.1	10.1	0.0	35.4	0.1	21.1	0.7
D*_int,oralBW5%ile, max_* (Internal dose, oral exposure, 5%ile bodyweight, maximum reported Phthalate concentration)	23.4	13.1	0.0	46.0	0.2	27.4	0.8
D*_int,oral Bwmean, mean_* (Internal dose, oral exposure, mean bodyweight, mean of reported Phthalate concentration)	1.1	1.0	0.0	5.6	0.0	4.1	0.2
D*_int,oralBW5%ile, mean_* (Internal dose, oral exposure, 5%ile bodyweight, mean of reported Phthalate concentration)	1.5	1.3	0.0	7.3	0.0	5.3	0.2
D*_int,oral Bwmean, med_* (Internal dose, oral exposure, mean bodyweight, median of reported Phthalate concentration)	0.0	0.0	0.0	0.0	0.0	0.5	0.1
D*_int,oralBW5%ile, med_* (Internal dose, oral exposure, 5%ile bodyweight, median of reported Phthalate concentration)	0.0	0.0	0.0	0.0	0.0	0.7	0.1
HQ*_BWmean, max_* (Hazard quotient at mean bodyweight and maximum phthalate concentration)	0.2	1.0	0.0	0.7	0.0	0.1	0.0
HQ*_BW5%ile, max_* (Hazard quotient at 5%ile bodyweight and maximum phthalate concentration)	0.2	1.3	0.0	0.9	0.0	0.2	0.0
HQ*_BWmean, mean_* (Hazard quotient at mean bodyweight and mean of phthalate concentration)	0.01	0.10	0.00	0.11	0.00	0.03	0.00
HQ*_BW5%ile, mean_* (Hazard quotient at 5%ile bodyweight and mean phthalate concentration)	0.0	0.1	0.0	0.1	0.0	0.0	0.0
HQ*_BWmean, med_* (Hazard quotient at mean bodyweight and median of phthalate concentration)	0.0	0.0	0.0	0.0	0.0	0.0	0.0
HQ*_BW5%ile, med_* (Hazard quotient at 5%ile bodyweight and median phthalate concentration)	0.0	0.0	0.0	0.0	0.0	0.0	0.0

**Table 5:** Calculated dose (µg/kg bw/day) and HQ for dermal exposure to phthalates at maximum, mean and median exposure concentrations.

Calculated Dose and HQ for Dermal Exposure	DIBP	DBP	BBP	DEHP	DNOP	DINP	DIDP
D*_int,dermalBWmean, max_* (Dose, dermal exposure, mean bodyweight, maximum reported Phthalate concentration)	6.6	3.7	0.0	13.0	0.0	7.8	0.2
D*_int,dermalBW5%ile, max_* (Dose, dermal exposure, 5%ile bodyweight, maximum reported Phthalate concentration)	8.6	4.8	0.0	16.9	0.1	10.1	0.3
D*_int,dermalBWmean, mean_* (Dose, dermal exposure, mean bodyweight, mean of reported Phthalate concentration)	0.4	0.4	0.0	2.1	0.0	1.5	0.1
D*_int,dermalBW5%ile, mean_* (Dose, dermal exposure, 5%ile bodyweight, mean of reported Phthalate concentration)	0.5	0.5	0.0	2.7	0.0	1.9	0.1
D*_int,dermalBWmean, med_* (Dose, dermal exposure, mean bodyweight, median of reported Phthalate concentration)	0.0	0.0	0.0	0.0	0.0	0.2	0.0
D*_int,dermalBW5%ile, med_* (Dose, dermal exposure, 5%ile bodyweight, median of reported Phthalate concentration)	0.0	0.0	0.0	0.0	0.0	0.2	0.1
HQ*_BWmean, max_*	0.1	0.4	0.0	0.3	0.0	0.1	0.0
HQ*_BW5%ile, max_*	0.1	0.5	0.0	0.3	0.0	0.1	0.0
HQ*_BWmean, mean_*	0.0	0.0	0.0	0.0	0.0	0.0	0.0
HQ*_BW5%ile, mean_*	0.0	0.0	0.0	0.1	0.0	0.0	0.0
HQ*_BWmean, med_*	0.0	0.0	0.0	0.0	0.0	0.0	0.0
HQ*_BW5%ile, med_*	0.0	0.0	0.0	0.0	0.0	0.0	0.0

**Table 6:** HI values for cumulative exposure to DEHP, BBP, DBP, and DIBP through oral and dermal routes, using maximum, mean, and median concentrations in toys.

HI for Phthalates Causing Developmental Toxicity	DIBP	DBP	BBP	DEHP	Cumulative
HI*_BW mean, max_* (Hazard index, mean bodyweight, maximum reported phthalate concentration)	0.2	1.4	0.0	1.0	2.6
HI*_BW5%ile, max_* (Hazard index, 5%ile bodyweight, maximum reported phthalate concentration)	0.3	1.8	0.0	1.3	3.4
HI*_BW mean, mean_* (Hazard index, mean bodyweight, mean of reported phthalate concentration)	0.0	0.1	0.0	0.2	0.3
HI*_BW5%ile, mean_* (Hazard index, 5%ile bodyweight, mean of reported phthalate concentration)	0.0	0.2	0.0	0.2	0.4
HI*_BW mean, med_* (Hazard index, mean bodyweight, median of reported phthalate concentration)	0.0	0.0	0.0	0.0	0.0
HI*_BW5%ile, med_* (Hazard index, 5%ile bodyweight, median of reported phthalate concentration)	0.0	0.0	0.0	0.0	0.0

**Table 7:** HI for hepatotoxicity from cumulative exposure to DINP, DIDP and DNOP via ingestion and dermal routes for maximum, mean and median concentrations.

HI for Phthalates Causing Hepatotoxicity	DNOP	DINP	DIDP	Cumulative
HI_BW mean, max_	0.0	0.2	0.0	0.2
HI_BW5%ile, max_	0.0	0.3	0.0	0.3
HI_BW mean, mean_	0.0	0.0	0.0	0.0
HI_BW5%ile, mean_	0.0	0.0	0.0	0.1
HI_BW mean, med_	0.0	0.0	0.0	0.0
HI_BW5%ile, med_	0.0	0.0	0.0	0.0

## Supplementary Materials

The following are available online at http://www.mdpi.com/1660-4601/15/2/200/s1, Table S1: Oral exposure and hazard quotient calculation for phthalates in children’s toys, Table S2: Dermal exposure and hazard quotient calculation for phthalates in children’s toys.
