# Supplementary material for: Analysis and Assessment of Exposure to Selected Phthalates Found in Children’s Toys in Christchurch, New Zealand
Source: Int J Environ Res Public Health. 2018 Jan 25;15(2):200. doi: 10.3390/ijerph15020200 (PMC5858269; doi:10.3390/ijerph15020200)
Supplement: Supplementary file 1 [file ijerph-15-00200-s001.docx]

Supplementary material

**Analysis and assessment of potential exposure from selected phthalates found in childrens toys in Christchurch, New Zealand.**

**Matthew James Ashworth^1*^, Andrew Chappell^1^, Ellen Ashmore^1^, Jefferson Fowles^2^**.

^1^ Institute of Environmental Science and Research (ESR) Ltd, New Zealand. Matthew.ashworth@esr.cri.nz

^2^ Tox-Logic Consulting, LLC. Petaluma, CA 94954, USA; tox-logic@hotmail.com

***** Correspondence: Matthew.ashworth@esr.cri.nz; Tel.: +64 (0)3 351 0022

Equation S1

$$D_{int,oral} =\left( \left( \frac{M \times S_{mouth} \times t \times n \times\frac{B_{oral}}{100}}{BW} \right) \times\frac{R_{phth}}{100} \right)$$

Where:

D_int,oral_ internal dose rate by oral route of specified phthalate (µg/kg bw/d)

M migration rate of specified phthalate from matrix (µg/cm^2^/h)

S_mouth_  surface area of infants open mouth (cm^2^)

t mouthing time (h)

n Frequency (d^-1^)

B_oral_ bioavailability via oral route (%, 100% assumed)

BW body weight (kg)

R_phth_ mass ratio of specified phthalate in matrix relative to experimental level in literature (43% by mass)

The R_phth_ value is derived by dividing the % mass fraction of specified phthalate by the phthalate reference material concentration value provided in literature (Babich 1998). This process allows the migration of the phthalate % mass fraction value to be normalised against the experimentally derived migration rate.

Equation S2

$$HQ = \frac{D_{int,oral}}{TDI}$$

Where:

HQ hazard quotient (unitless)

TDI tolerable daily intake (µg/kg bw/d)

Equation S3

$$D_{int,derm} = \left( \frac{R_{derm} \times S_{derm} \times t \times n}{BW} \right) \times\frac{R_{phth}}{100}$$

Where:

D_int,derm_ internal dose via the dermal route (µg/kg bw/d)

R_derm_ dermal absorption rate of specified phthalate in skin (µg/cm^2^/h)

S_derm_ surface area of child’s lips and hands (cm^2^)

t contact time (h)

n frequency (d^-1^)

BW body weight (kg)

R_phth_ mass ratio of specified phthalate in matrix relative to experimental level in literature (40.4% by mass)

Table S1 Oral exposure and hazard quotient calculation for phthalates in children’s toys.

|  | DIBP | DBP | BBP | DEHP | DNOP | DINP | DIDP |
| --- | --- | --- | --- | --- | --- | --- | --- |
| M | 26.03 | 26.03 | 26.03 | 26.03 | 26.03 | 26.03 | 26.03 |
| S_mouth_ (cm^2^) | 10 | 10 | 10 | 10 | 10 | 10 | 10 |
| t x n (hours/day) | 0.8 | 0.8 | 0.8 | 0.8 | 0.8 | 0.8 | 0.8 |
| B_oral_ | 1 | 1 | 1 | 1 | 1 | 1 | 1 |
| BW_mean_ (kg) | 7.4 | 7.4 | 7.4 | 7.4 | 7.4 | 7.4 | 7.4 |
| BW_5th %ile_ (kg) | 5.7 | 5.7 | 5.7 | 5.7 | 5.7 | 5.7 | 5.7 |
| R_phth, max_ | 0.64 | 0.36 | 0.00 | 1.26 | 0 | 0.75 | 0.02 |
| R_phth, mean_ | 0.0398 | 0.0356 | 0.0002 | 0.2005 | 0.0009 | 0.1442 | 0.0058 |
| R_phth, median_ | 0.0009 | 0.0007 | 0.0002 | 0.0002 | 0.0002 | 0.0181 | 0.0040 |
| D_int,oral Bwmean, max_ | 18.1 | 10.1 | 0.0 | 35.4 | 0.0 | 21.1 | 0.7 |
| D_int,oralBW5%ile, max_ | 23.4 | 13.1 | 0.0 | 46.0 | 0.0 | 27.4 | 0.8 |
| D_int,oral Bwmean, mean_ | 1.1 | 1.0 | 0.0 | 5.6 | 0.0 | 4.1 | 0.2 |
| D_int,oralBW5%ile, mean_ | 1.5 | 1.3 | 0.0 | 7.3 | 0.0 | 5.3 | 0.2 |
| D_int,oral Bwmean, med_ | 0.0 | 0.0 | 0.0 | 0.0 | 0.0 | 0.5 | 0.1 |
| _Dint,oralBW5%ile, med_ | 0.0 | 0.0 | 0.0 | 0.0 | 0.0 | 0.7 | 0.1 |
| TDI | 100 | 10 | 500 | 50 | 150 | 150 | 150 |
| HQ _Bwmean, max_ | 0.2 | 1.0 | 0.0 | 0.7 | 0.0 | 0.1 | 0.0 |
| HQ _BW5%ile, max_ | 0.2 | 1.3 | 0.0 | 0.9 | 0.0 | 0.2 | 0.0 |
| HQ _Bwmean, mean_ | 0.01 | 0.10 | 0.00 | 0.11 | 0.00 | 0.03 | 0.00 |
| HQ _BW5%ile, mean_ | 0.0 | 0.1 | 0.0 | 0.1 | 0.0 | 0.0 | 0.0 |
| HQ _Bwmean, med_ | 0.0 | 0.0 | 0.0 | 0.0 | 0.0 | 0.0 | 0.0 |
| HQ _BW5%ile, med_ | 0.0 | 0.0 | 0.0 | 0.0 | 0.0 | 0.0 | 0.0 |

Table S2 Dermal exposure and hazard quotient calculation for phthalates in children’s toys.

|  | DIBP | DBP | BBP | DEHP | DNOP | DINP | DIDP |
| --- | --- | --- | --- | --- | --- | --- | --- |
| R_derm_ (ug/cm^2^/h) | 0.24 | 0.24 | 0.24 | 0.24 | 0.24 | 0.24 | 0.24 |
| S_derm_ (cm^2^) | 100 | 100 | 100 | 100 | 100 | 100 | 100 |
| t x n (h/d) | 3 | 3 | 3 | 3 | 3 | 3 | 3 |
| BW_mean_ (kg) | 7.4 | 7.4 | 7.4 | 7.4 | 7.4 | 7.4 | 7.4 |
| BW_5th %ile_ (kg) | 5.7 | 5.7 | 5.7 | 5.7 | 5.7 | 5.7 | 5.7 |
| R_phth, max_ | 0.68 | 0.38 | 0.00 | 1.34 | 0.00 | 0.80 | 0.02 |
| R_phth, mean_ | 0.04 | 0.04 | 0.00 | 0.21 | 0.00 | 0.15 | 0.01 |
| R_phth, median_ | 0.00 | 0.00 | 0.00 | 0.00 | 0.00 | 0.02 | 0.00 |
| D_int,dermalBWmean, max_ | 6.6 | 3.7 | 0.0 | 13.0 | 0.0 | 7.8 | 0.2 |
| D_int,dermalBW5%ile, max_ | 8.6 | 4.8 | 0.0 | 16.9 | 0.0 | 10.1 | 0.3 |
| D_int,dermalBWmean, mean_ | 0.4 | 0.4 | 0.0 | 2.1 | 0.0 | 1.5 | 0.1 |
| D_int,dermalBW5%ile, mean_ | 0.5 | 0.5 | 0.0 | 2.7 | 0.0 | 1.9 | 0.1 |
| D_int,dermalBWmean, med_ | 0.0 | 0.0 | 0.0 | 0.0 | 0.0 | 0.2 | 0.0 |
| D_int,dermalBW5%ile, med_ | 0.0 | 0.0 | 0.0 | 0.0 | 0.0 | 0.2 | 0.1 |
| TDI | 100 | 10 | 500 | 50 | 150 | 150 | 150 |
| HQ _BWmean, max_ | 0.1 | 0.4 | 0.0 | 0.3 | 0.0 | 0.1 | 0.0 |
| HQ _BW5%ile, max_ | 0.1 | 0.5 | 0.0 | 0.3 | 0.0 | 0.1 | 0.0 |
| HQ _BWmean, mean_ | 0.0 | 0.0 | 0.0 | 0.0 | 0.0 | 0.0 | 0.0 |
| HQ _BW5%ile, mean_ | 0.0 | 0.0 | 0.0 | 0.1 | 0.0 | 0.0 | 0.0 |
| HQ _BWmean, med_ | 0.0 | 0.0 | 0.0 | 0.0 | 0.0 | 0.0 | 0.0 |
| HQ _BW5%ile, med_ | 0.0 | 0.0 | 0.0 | 0.0 | 0.0 | 0.0 | 0.0 |

References

Babich MA. 1998. The risk of chronic toxicity associated with exposure to di-isononyl phthalate (dinp) in children's products. Available: <http://www.docstoc.com/docs/48047842/The-Risk-of-Chronic-Toxicity-Associated-with-Exposure-to> [accessed 29 October 2014.
